# Pharmacokinetics and Efficacy of Topically Applied Nonsteroidal Anti-Inflammatory Drugs in Retinochoroidal Tissues in Rabbits

**DOI:** 10.1371/journal.pone.0096481

**Published:** 2014-05-05

**Authors:** Tetsuo Kida, Seiko Kozai, Hiroaki Takahashi, Mitsuyoshi Isaka, Hideki Tokushige, Taiji Sakamoto

**Affiliations:** 1 Research Laboratories for Drug Development, Senju Pharmaceutical Co., Ltd., Kobe, Japan; 2 Department of Ophthalmology, Kagoshima University Graduate School of Medical and Dental Sciences, Kagoshima, Japan; University of Florida, United States of America

## Abstract

**Purpose:**

To evaluate the pharmacokinetics and efficacy of topically applied nonsteroidal anti-inflammatory drugs (NSAIDs) in the retinochoroidal tissues of rabbits.

**Methods:**

The cyclooxygenase (COX) inhibitory activity of diclofenac, bromfenac, and amfenac, an active metabolite of nepafenac, were determined using human-derived COX-1 and COX-2. Each of the three NSAIDs was applied topically to rabbits, and after 0.5 to 8 hrs, the concentration of each drug in the aqueous humor and the retinochoroidal tissues was measured by liquid chromatography-tandem mass spectrometry. The pharmacokinetics of the drugs in the tissues after repeated doses as is done on patients was calculated by a simulation software. The inhibitory effect of each NSAID on the breakdown of the blood-retinal barrier was assessed by the vitreous protein concentration on concanavalin A-induced retinochoroidal inflammation in rabbits.

**Results:**

The half-maximal inhibitory concentration (IC_50_) of diclofenac, bromfenac, and amfenac was 55.5, 5.56, and 15.3 nM for human COX-1, and 30.7, 7.45, and 20.4 nM for human COX-2, respectively. The three NSAIDs were detected in the aqueous humor and the retinochoroidal tissue at all-time points. Simulated pharmacokinetics showed that the levels of the three NSAIDs were continuously higher than the IC_50_ of COX-2, as an index of efficacy, in the aqueous humor, whereas only the bromfenac concentration was continuously higher than the IC_50_ at its trough level in the retinochoroidal tissues. The intravitreous concentration of proteins was significantly reduced in rabbits that received topical bromfenac (*P* = 0.026) but not the other two NSAIDs.

**Conclusions:**

Topical bromfenac can penetrate into the retinochoroidal tissues in high enough concentrations to inhibit COX-2 and exerts its inhibitory effect on the blood-retinal barrier breakdown in an experimental retinochoroidal inflammation in rabbits. Topical bromfenac may have a better therapeutic benefit than diclofenac and nepafenac for retinochoroidal inflammatory diseases.

## Introduction

Angiogenic retinal diseases including neovascular age-related macular degeneration (nAMD) are the leading causes of blindness in the elder populations in developed countries [1]–[3]. At present, anti-vascular endothelial growth factor (VEGF) treatment is becoming the gold standard therapy for nAMD and monthly injections have been found to be most effective in improving and maintaining the maximum vision in eyes with nAMD [4], [5]. Despite the improvement of vision by this treatment, there remain significant needs for better treatments. The injection of anti-VEGF agents is costly and requires frequent office visits. This protocol places a significant burden on patients, their family members, and physicians [6].

Although the exact mechanism for the development of nAMD is not fully understood, it is accepted that inflammation plays a substantial role in the disease processes [7]. Thus, it is reasonable to use anti-VEGF therapy with adjunctive anti-inflammatory therapy which might reduce the treatment burden of anti-VEGF agents.

To inhibit or control inflammation, topical steroids are the mainstay therapy in ophthalmology [8]. However, steroids have serious adverse effects such as an elevation of the intraocular pressure and progression of cataracts [9]. Nonsteroidal anti-inflammatory drugs (NSAIDs) have proven to be safe and effective alternatives to steroids in the management of ocular inflammations [10]. Currently, these drugs are used topically for the inhibition of intraoperative miosis, management of postoperative inflammation, and other inflammatory conditions [11]–[13]. Although it is not at the clinical level, 0.5% indomethacin ophthalmic suspension containing hydroxypropylmethylcellulose (Indom, Alfa Intes Srl, Casoria, Italy) or 0.1% indomethacin ophthalmic solution with hydroxypropyl-β-cyclodextrin (Indocollirio, Bausch & Lomb IOM SpA, Vimodrone, Italy) attain high concentrations in ocular tissues in rabbits [14].

Bromfenac, 2-amino-3-(4-bromobenzoyl) phenylacetic acid, is a NSAID which is widely used as an ophthalmic solution for the treatment of postoperative ocular inflammation and pain following cataract surgery, and/or inflammatory diseases of anterior and outer eye segments [15], [16]. Recently, we found that topical bromfenac inhibited the laser-induced choroidal neovascularization in rats [17]. In humans, Gomi et al. reported that adjunctive use of topical bromfenac could reduce the frequency of intravitreous ranibizumab [18]. These results indicated the potential benefit of topical bromfenac for retinochoroidal inflammatory diseases.

Although bromfenac has very good intraocular penetration into the aqueous humor in human [19], detailed pharmacokinetics for retinochoroidal tissue has not been determined. In rabbits, Baklayan et al. have reported on the pharmacokinetic profiles of ocular tissues by using topical radiolabeled bromfenac [20], however, the relationship of the pharmacokinetics and efficacy in the target tissues has not been determined. In addition, the pharmacokinetic profiles of bromfenac after repeated doses such as is done in the usual clinical condition in comparison with the other NSAID eye drops have not been determined. Furthermore, the pharmacokinetic profiles determined by radiolabeled compounds provide limited information because the profiles may potentially be affected by the related compounds having radioactivity, such as the metabolites and degradation products.

Thus, the purpose of this study was to determine the drug levels of bromfenac, diclofenac, and nepafenac in the retinochoroidal tissues of rabbits by liquid chromatography-tandem mass spectrometry (LC-MS/MS) after a single topical administration of these NSAID eye drops. We also compared the simulated pharmacokinetic profiles of topical bromfenac to the other NSAID eye drops in the repeated dosing regimen with a theoretical model. In addition, the inhibitory effect of these topically applied NSAIDs on the breakdown of blood-retinal barrier was studied in rabbits.

## Materials and Methods

### Ethics Statement

Human blood samples were obtained from Taipei Blood Center (Taipei, Taiwan) according to the principles expressed in the Declaration of Helsinki, and all donors had signed a written informed consent. The procedures for the assay protocol were approved by the Institutional Biosafety Committee, which is functionally equivalent to the Institutional Review Board, of MDS Pharma Services (Taipei, Taiwan). All animal experiments were conducted in accordance with the recommendations in the Guide for the Care and Use of Laboratory Animals of the National Institutes of Health, and the Association for Research in Vision and Ophthalmology (ARVO) Statement for the Use of Animals in Ophthalmic and Vision Research. The protocols were approved by the Institutional Animal Care and Use Committees of Senju Pharmaceutical Co., Ltd. (Permit Number: 20120130-01) and Shin Nippon Biomedical Laboratories, Ltd. (Kagoshima, Japan, Permit Number: 12024). All efforts were made to minimize pain and suffering.

### Animals

Male Japanese white rabbits (Kbl:JW) weighing approximately 2.0 kg were obtained from Kitayama Labes Co., Ltd. (Ina, Japan), and male Dutch-Belted rabbits (Kbt:Dutch) weighing approximately 2.0 kg were obtained from Biotek Co., Ltd. (Tosu, Japan). The animals were housed in a controlled temperature (23°C±2°C) and humidity (55%±10%) room under a 12-hour light/12-hour dark cycle under specific pathogen-free conditions. Animals were fed a standard chow diet (100 g/day) and given sterilized water to drink ad libitum.

### Reagents

**Figure 1:**
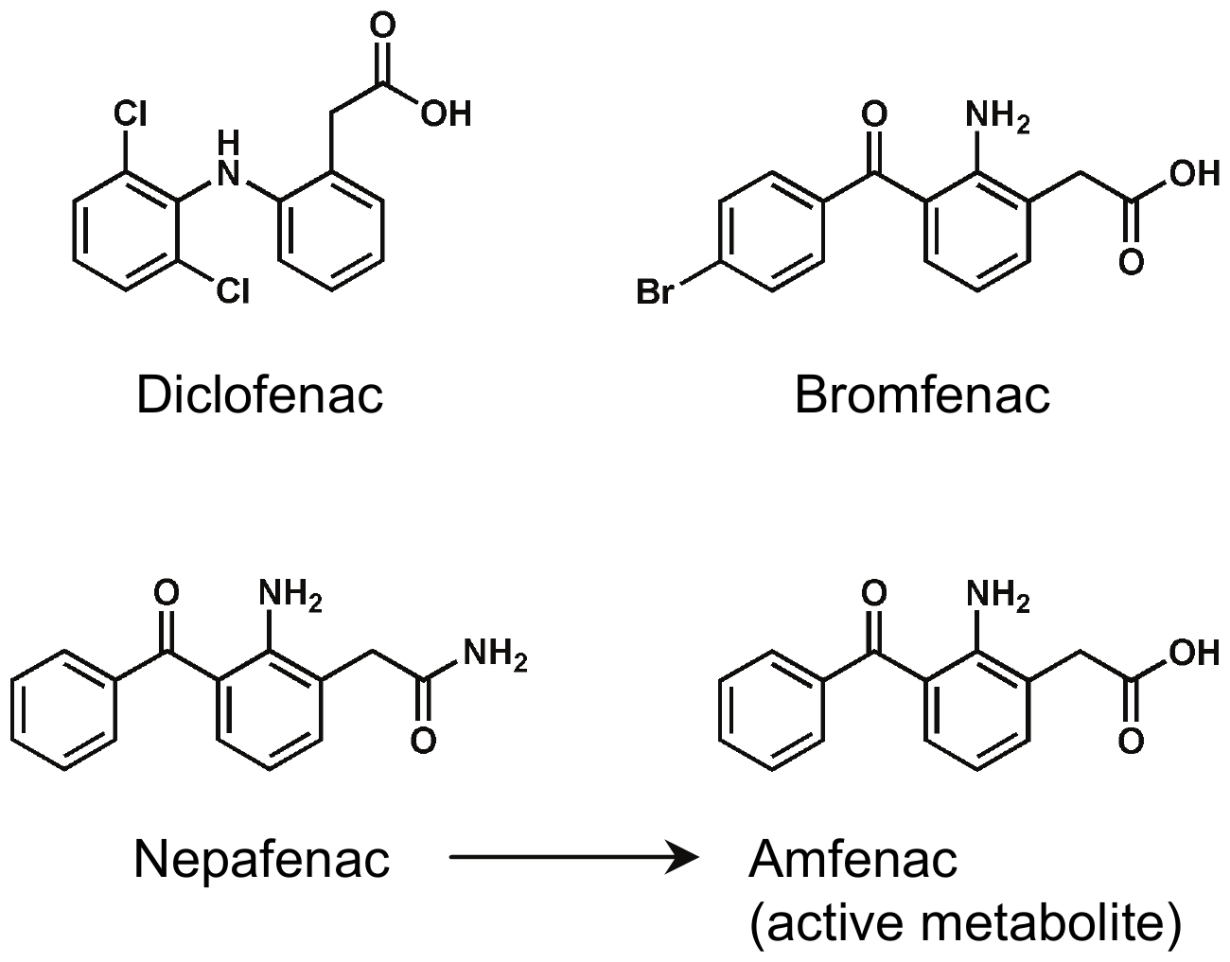
Chemical structures of diclofenac, bromfenac, nepafenac, and amfenac.

For the in vitro experiments, sodium diclofenac was purchased from Sigma-Aldrich Co. (St. Louis, MO), sodium bromfenac was provided by Senju Pharmaceutical Co., Ltd. (Osaka, Japan), and sodium amfenac was synthesized by Torcan Chemical Ltd. (Ontario, Canada). For the in vivo experiments, 0.1% diclofenac sodium ophthalmic solution (Diclod, Wakamoto Co., Ltd., Tokyo, Japan), 0.1% bromfenac sodium hydrate ophthalmic solution, identical to 0.09% bromfenac ophthalmic solution, (Bronuck, Senju Pharmaceutical Co., Ltd.), and 0.1% nepafenac ophthalmic suspension (Nevanac, Alcon Japan Ltd., Tokyo, Japan) were obtained from commercial sources (Figure 1).

### Cyclooxygenase Inhibitory Activity

The inhibition of cyclooxygenase (COX)-1 and COX-2 activity by diclofenac, bromfenac, and amfenac was determined by measuring the production of prostaglandin (PG) E_2_ using an enzyme immunoassay (EIA) kit (Prostaglandin E_2_ Biotrak EIA system, GE Healthcare, Buckinghamshire, UK). The endogenous human COX-1 inhibitory activity was assayed by a published method [21] with a slight modification, using venous blood of healthy volunteers with a written informed consent (obtained from Taipei Blood Center, Taipei, Taiwan). Test samples (0.3 to 1000 nM) and vehicle (1% dimethyl sulfoxide in the final assay) were pre-incubated with washed human platelets (5×10^7^/mL) in modified buffer (Hanks' balanced salt solution with 15 mM HEPES, pH 7.4) for 15 minutes at 37°C. Arachidonic acid (100 µM, Cayman Chemical Co., Ann Arbor, MI) was added to initiate the reaction, and samples were incubated for an additional 15 minutes at 37°C. At the end of the incubation period, the reaction was terminated by the addition of 1 N HCl. Aliquots were then removed to determine the amount of PGE_2_ produced with the EIA kit. The amount of PGE_2_ was spectrophotometrically measured in the range of 50–6400 pg/mL at 450 nm according to the instructions of the kit using a competitive microtiter-based immunoassay method. Tests were performed with 7 concentrations of each drug to construct a dose-response curve and to determine the concentration causing a half-maximal inhibition (IC_50_) relative to the control values. Each series of concentrations was run in triplicate for each drug. In each experiment, the reference compound for COX-1, indomethacin (Sigma-Aldrich, IC_50_ = 32.8 nM), was tested concurrently. The IC_50_ of each drug was obtained by a 4-parameter logistic fitting method using GraphPad Prism, ver 4.02 software (GraphPad Software, Inc., La Jolla, CA). The equation for a 4-parameter logistic fitting was,
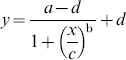



where a is the maximum value of the sigmoid curve; b is the Hill slope; c is (a + d)/2 (or IC_50_); and d is the lowest value of the sigmoid curve.

Human recombinant COX-2 isolated from baculovirus-infected Sf21 cells (Sigma-Aldrich) was used for this assay. Test articles (0.1–1000 nM) and vehicle (1% dimethyl sulfoxide in the final assay) were pre-incubated with buffer (100 mM Tris-HCl, pH 7.7) containing 0.11 U COX-2, 1 mM glutathione, 1 µM hematin, and 0.5 mM phenol for 15 minutes at 37°C. Arachidonic acid (0.3 µM) was added to initiate the reaction, and samples were incubated for an additional 5 minutes at 37°C. At the end of the incubation period, the reaction was terminated by adding 1 N HCl. The amount of PGE_2_ produced was determined with the EIA kit, and the IC_50_ of each drug for COX-2 was obtained as described. Rofecoxib (Sigma-Aldrich, IC_50_ = 202 nM) was used as the reference compound for COX-2 enzyme.

### Ocular Distribution of NSAIDs after Single Topical Instillation in Rabbits

Thirty rabbits (Kbl:JW) were divided into 5 equal groups to study at 0.5, 1, 2, 4, and 8 hours after topical NSAID application. One of the three NSAID eye drops (30 µL: 0.1% diclofenac sodium ophthalmic solution, 0.1% bromfenac sodium hydrate ophthalmic solution, or 0.1% nepafenac ophthalmic suspension) was given topically to one eye of each rabbit. Another eye drop was instilled in the contralateral eye of the rabbit in the same way. After the indicated times, the rabbits were exsanguinated under deep anesthesia by intravenous injection of sodium pentobarbital (30 mg/mL) through an ear vein. Then, the eyes were enucleated and immediately washed with physiological saline. After collecting aqueous humor in a syringe, the eyes were frozen in liquid nitrogen. The retinochoroidal tissues were carefully dissected from the frozen eyes and weighed. The retinochoroidal tissue was homogenized with methanol in a final dilution 20 times the volume of the tissue sample. The homogenate and the aqueous humor were kept frozen at -80°C until each analysis.

The collected aqueous humor and retinochoroidal homogenate were purified and concentrated with a solid-phase extraction kit (Oasis HLB 96-well μElution Plate, Waters Corp., Milford, MA). Briefly, the aqueous humor (25 µL) was mixed with an internal reference standard (20 µL), diclofenac-d4, dissolved in methanol, and diluted in 255 µL of ultra-pure water. An aliquot (15 µL) was diluted in 285 µL of ultra-pure water and applied to the μElution Plate. After washing with 5% methanol, a sample was eluted with 100 µL of methanol and diluted in 150 µL of ultra-pure water. In the retinochoroidal homogenate, an aliquot (50 µL) was mixed with diclofenac-d4 solution (20 µL) and evaporated to dryness. The dried residue was redissolved in 300 µL of ultra-pure water, and it was applied to the plate. The sample was purified and concentrated in the same way. Then, the concentration of each drug in the samples extracted from aqueous humor or the retinochoroidal homogenate was measured by LC-MS/MS (QTRAP 5500, AB SCIEX, Framingham, MA) with a reverse-phase column (ACQUITY UPLC BEH C18, 2.1×50 mm, 1.7 µm, Waters Corp.). The quantification was done by multiple reaction monitoring mode (positive), with a Q1/Q3 (m/z) combination of 334/288, 255/210, 256/210, 296/214, and 302/256 for bromfenac, nepafenac, amfenac, diclofenac, and dicrofenac-d4, respectively. Electrospray with positive ionization was used (detailed analytical condition of LC-MS/MS is presented in Appendix S1). When the concentration was less than the quantification limit (1 ng/mL or 1 ng/g), it was expressed as not detected (ND) and assigned a value of 0 in the pharmacokinetic analyses. The specificity, linearity, and reproducibility of the measurements and the freeze-thaw stability of the samples were confirmed.

### Pharmacokinetic Analysis of NSAID Concentration in Ocular Tissues

The pharmacokinetic parameters were calculated by a non-compartmental model using Phoenix WinNonlin ver 6.1 (Pharsight Corporation, St. Louis, MO) based on the results of ocular pharmacokinetics after a single dose, and the pharmacokinetics of repeated dose was simulated for each drug using the same software. The interval of administration was determined by the general clinical use of each drug. For example, the interval was 8 hours for diclofenac and nepafenac because they are used clinically 3 times/day. The interval was 12 hours for bromfenac because it is used clinically twice/day. The IC_50_ of each NSAID for human COX-2 was taken as an index of the anti-inflammatory activity in the tissue, and it was compared to its simulated pharmacokinetic profile after repeated administration.

### Concanavalin A-induced Intraocular Inflammation in Rabbits

The concentration of leaked proteins in the vitreous of eyes with concanavalin A (ConA)-induced intraocular inflammation was used to determine whether a breakdown of the blood-retinal barrier had occurred. Thirty-seven rabbits (Kbt:Dutch) were randomly divided into 6 groups of 6 each with one group with 7 rabbits. One group received sham treatment (n = 6, Group 1) and was the negative control group. Group 1 and Group 2 (n = 6) received physiological saline (Otsuka Normal Saline, Otsuka Pharmaceutical Factory Inc., Tokushima, Japan), Group 3 received 0.1% diclofenac sodium ophthalmic solution (n = 7), Group 4 received 0.1% bromfenac sodium hydrate ophthalmic solution (n = 6), Group 5 received 0.1% nepafenac ophthalmic suspension (n = 6), and Group 6 received subcutaneous indomethacin as a positive control (n = 6). Each of the rabbits in Groups 1 through 5 had 50 µL of a topical application of the NSAID or saline 3 times/day for 4 days. Six rabbits (Group 6) received a subcutaneous injection of indomethacin (20 mg/kg, Nacalai Tesque, Inc., Kyoto, Japan) dissolved in 4% NaHCO_3_ once a day. Twenty-four hours after the first administration, rabbits were anesthetized by an intramuscular injection of a 3∶1 mixture of 5% ketamine (Ketalar for intramuscular injection 500 mg, Daiichi Sankyo Co., Ltd., Tokyo, Japan) and 2% xylazine (Rompun, Bayer HealthCare AG, Monheim, Germany) at 1 mL/kg. Then, retinal inflammation was induced by the method of Kapin et al. with slight modifications [22]. Briefly, 20 µL of Con A (0.25 mg/mL, Nacalai Tesque, Inc.) dissolved in physiological saline was injected into the vitreous body of the right eye in 31 rabbits (Groups 2 to 6). Six rabbits (Group 1 as sham treatment) received 20 µL of saline intravitreally instead of Con A. Seventy-two hours after the Con A or saline injection, the animals were euthanized with an overdose of intravenous pentobarbital. Then, the eyes were enucleated and placed in ice-cold PBS. The globe was dissected on ice to collect tissues from the posterior segment of the eye. A circumflex incision was made 1 to 3 mm posterior to the limbus, and the vitreous body was separated from the anterior segment by gently pulling the sclera. The vitreous gel was cut free from the anterior segment, placed in 40 µL of a Halt protease inhibitor cocktail (Thermo Fisher Scientific K.K., Yokohama, Japan) and frozen on dry ice. All samples were stored at -80°C until analysis.

Protein analysis was performed using a Pierce BCA Protein Assay Reagent kit (Thermo Fisher Scientific K.K.) according to a standard protocol. Bovine serum albumin (2 mg/mL) was used as the reference standard for the assays. The optical density of the reference standards and the 10-fold diluted samples was measured in the range of 20–2000 µg/mL at 562 nm with a spectrophotometer (Powerscan HT, DS Pharma Biomedical Co., Ltd, Osaka, Japan). The protein concentration in the samples was calculated from a standard curve and corrected for any dilution.

### Statistical Analyses

For statistical analysis of Con A-induced intraocular inflammation, the comparisons of the negative control eyes (sham group) and Con A-treated and followed by saline administration eyes (saline group) was performed by the Student's *t* test (1-sided). In the Con A-treated eyes, the comparison between eyes with saline administration (saline group) and those with drug administration was analyzed by Dunnett's test (1-sided). A *P*-value <0.05 was considered statistically significant. All statistical analyses were performed using JMP 7.0 software (SAS Institute Inc., Cary, NC).

## Results

### COX Inhibitory Activities of NSAIDs

The IC_50_ of bromfenac on human COX-1 was 5.56 nM followed by amfenac at 15.3 nM and diclofenac at 55.5 nM. The IC_50_ of bromfenac on human COX-2 was 7.45 nM followed by amfenac at 20.4 nM and diclofenac at 30.7 nM (Table 1). The inhibitory activities of bromfenac on COX-1 and COX-2 were 10 times and 4.1 times more potent than diclofenac, and 2.8 times and 2.7 times more potent than amfenac. The selectivity ratios of IC_50_ of human COX-2 to COX-1 were 0.55 for diclofenac, 1.3 for bromfenac, and 1.3 for amfenac (Table 1).

**Table 1:** Cyclooxygenase (COX) inhibitory activities of diclofenac, bromfenac, and amfenac.

	IC_50_ (nM), [95% Confidence interval (nM)]		
Compounds	COX-1	COX-2	Ratio of IC_50_
Diclofenac	55.5, [19.6–158]	30.7, [19.1–50.5]	0.55
Bromfenac	5.56, [3.55–8.83]	7.45, [4.58–12.0]	1.3
Amfenac	15.3, [10.6–22.2]	20.4, [14.7–27.9]	1.3

Ratio of IC_50_: IC_50_ for COX-2/IC_50_ for COX-1

The IC_50_ of indomethacin was used as a reference compound for COX-1, and its IC_50_ was 32.8 nM and that of rofecoxib for COX-2 was 202 nM.

### Ocular Distribution of NSAIDs after Single Topical Instillation

**Figure 2:**
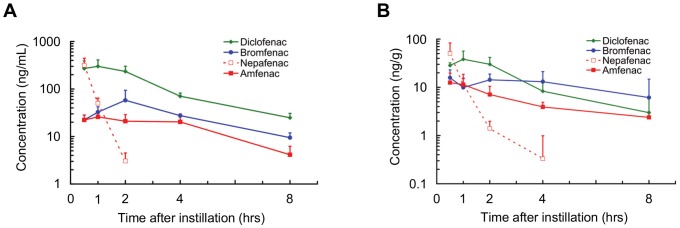
Comparison of ocular tissue concentrations of topically applied NSAIDs. Concentrations of diclofenac, bromfenac, nepafenac, and amfenac in the aqueous humor (A) and retinochoroidal tissue (B) after a single topical application in rabbits. Thirty microliters of 0.1% bromfenac sodium ophthalmic solution, 0.1% diclofenac ophthalmic solution, or 0.1% nepafenac ophthalmic suspension were topically applied to the rabbit eye. Samples of aqueous humor and choroid/retina were collected at the designated times. Amfenac is an active metabolite of nepafenac. Each value represents the mean + standard deviation (n = 4/time point).

The aqueous humor concentration of nepafenac was highest at 30 minutes, and it decreased rapidly with increasing time, whereas amfenac, which was enzymatically hydrolyzed from nepafenac, was present for a considerably longer time in the aqueous humor (Figure 2A). The maximum concentration (C_max_) of diclofenac was 300 ng/mL at 1 hour, that for bromfenac was 57.8 ng/mL at 2 hours, and that amfenac was 25.9 ng/mL at 1 hour (Table 2). Nepafenac was rapidly dispersed in the aqueous humor, viz., time to maximum concentration (T_max_) was 0.5 hr, however the presence of bromfenac was relatively long-lasting (T_max_, 2 hr). The clearance pattern from the aqueous humor for the 3 NSAIDs was very similar (Figure 2A and Table 2).

**Table 2:** Pharmacokinetic parameters of single instillation of NSAID eye drops in aqueous humor.

	C_max_ (ng/mL)*	T_max_ (hr)	T_1/2_ (hr)	k_el_ (hr^−1^)	AUC_0–8_ (ng·hr/mL)^†^	MRT (hr)
Diclofenac	300±110	1	1.94	0.358	972±44	2.30
Bromfenac	57.8±36.1	2	2.34	0.296	224±33	3.06
Amfenac	25.9±7.4	1	2.39	0.290	131±11	3.03

AUC_0–8_: The area under the aqueous humor drug concentration-time curve from 0 to 8 hours, k_el_: elimination rate constant, MRT: mean residence time, *: Mean ± S.D., †: Mean ± SEM.

Nepafenac was converted to amfenac quickly and amfenac reached the retinochoroidal tissues quickly (T_max_, 0.5 hr; Figure 2B). The C_max_ of diclofenac, bromfenac, and amfenac in the retinochoroidal tissue were 38.2 ng/g at 1 hour, 15.8 ng/g at 30 minutes, and 12.5 ng/g at 30 minutes, respectively (Table 3). The drug clearance of diclofenac was fast at the elimination half-life (T_1/2_), 1.89 hr, whereas the clearances of bromfenac and amfenac were relatively slow at the T_1/2_, 4.69 hr and 4.00 hr, respectively (Figure 2B and Table 3).

**Table 3:** Pharmacokinetic parameters of single instillation of NSAID eye drops in retinochoroidal tissues.

	C_max_ (ng/g)*	T_max_ (hr)	T_1/2_ (hr)	k_el_ (hr^−1^)	AUC_0–8_ (ng·hr/g)^†^	MRT (hr)
Diclofenac	38.2±18.1	1	1.89	0.366	119±27	2.30
Bromfenac	15.8±7.2	0.5	4.69	0.148	88.4±9.1	3.51
Amfenac	12.5±7.2	0.5	4.00	0.173	41.9±3.4	2.82

AUC_0–8_: The area under the retinochoroidal drug concentration-time curve from 0 to 8 hours, k_el_: elimination rate constant, MRT: mean residence time, * Mean ± S.D., † Mean ± SEM.

### Pharmacokinetic Analysis of Simulated NSAID Concentrations in Aqueous Humor and Retinochoroidal Tissues Following Repeat Dose Administration

**Figure 3:**
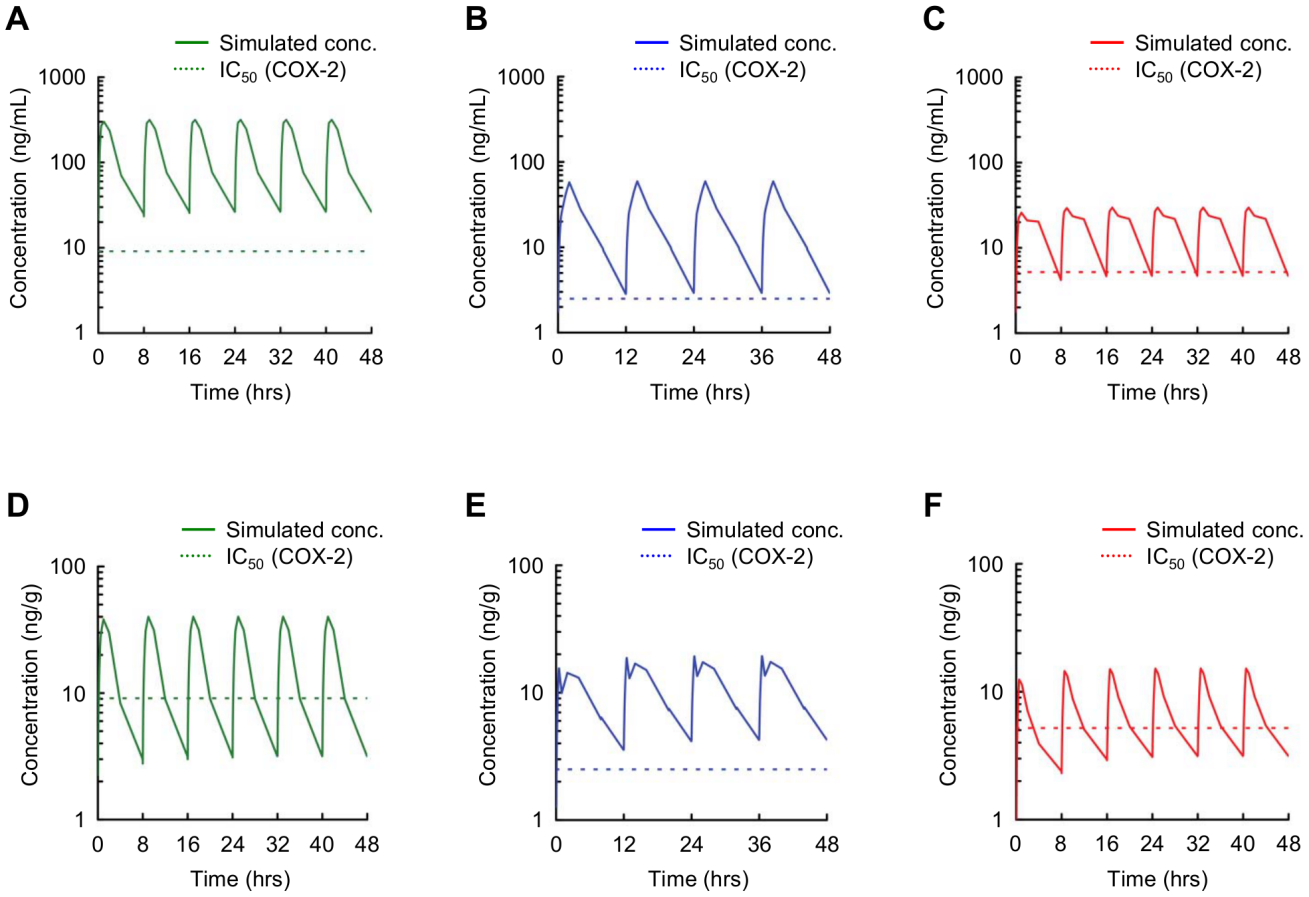
The simulated drug concentrations in aqueous humor and retina/choroid with the IC_50_ for COX-2. The pharmacokinetic profiles of diclofenac (A) and amfenac (C) in the aqueous humor were calculated on the basis of a three/day dosing at an interval of eight hours, and the profile of bromfenac (B) was calculated on the basis of a two/day dosing at an interval of twelve hours. The retinochoroidal pharmacokinetic profiles of diclofenac (D), bromfenac (E) and amfenac (F) were also calculated in the same manner. The simulated concentrations (solid line) of diclofenac (A) and bromfenac (B) in the aqueous humor were higher than their corresponding IC_50_ values of the NSAIDs for COX-2 (dotted line). The concentrations of amfenac (C) were almost always higher than the IC_50_ of COX-2 although it was slightly less than the IC_50_ at the trough level. In retinochoroidal tissues, the simulated concentration of bromfenac (E) with a two/day dosing was higher than the IC_50_ value for COX-2, whereas the concentrations of diclofenac (D) and amfenac (F) with a three/day dosing were lower than the corresponding IC_50_ values at the troughs. Each value of the IC_50_ for COX-2 indicated in the figures is referred in Table 1.

The concentrations for the transition models were calculated based on the results of a single dose. The interval between instillations was determined by the interval usually used in the clinic for each drug. The concentrations of diclofenac and bromfenac in the aqueous humor were found to be continuously higher than the IC_50_ of COX-2 as an index of efficacy. The concentrations of amfenac were almost always higher than the IC_50_ of COX-2 although it was slightly less than the IC_50_ at the trough level (Figure 3A–C).

The peak levels of diclofenac and amfenac exceeded the IC_50_ of COX-2 in the retinochoroidal tissue, however it was less than the IC_50_ of COX-2 at the trough level. On the other hand, the concentration of bromfenac was always higher than the IC_50_ of COX-2 with a twice/day dosing regimen (Figure 3D–F).

### Effects of NSAIDs on Con A-induced Intraocular Inflammation in Rabbits

**Figure 4:**
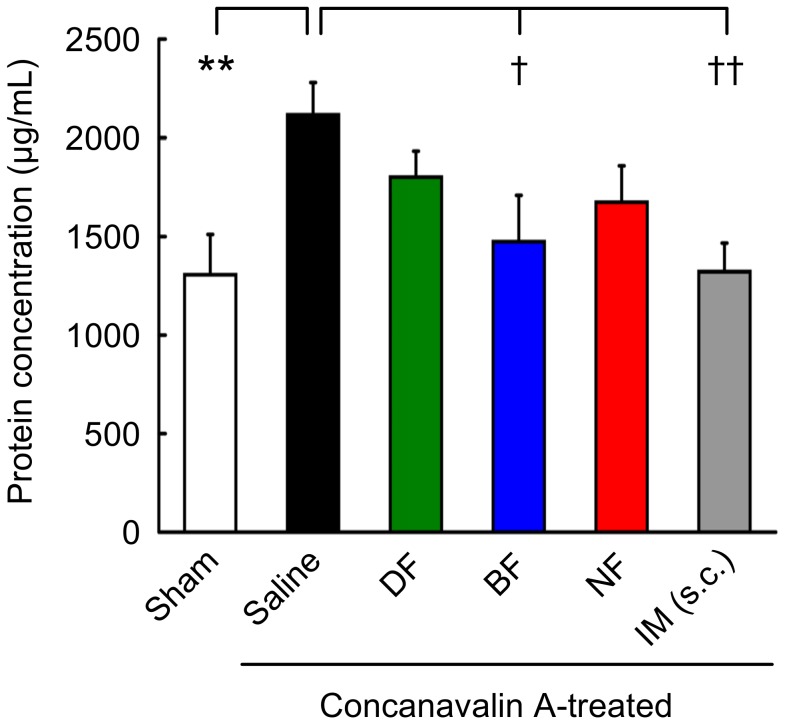
Inhibitory effect of NSAID eye drops on the concanavalin A-induced retinochoroidal inflammation in rabbits. Rabbits received 50 µL of 0.1% diclofenac ophthalmic solution (DF), 0.1% bromfenac sodium hydrate ophthalmic solution (BF), 0.1% nepafenac ophthalmic suspension (NF), physiological saline (Saline) three times a day on days -1, 0 (the day of intravitreal concanavalin A injection), 1, and 2. For positive controls, rabbits were subcutaneously injected with indomethacin (IM, 20 mg/kg) once a day during the same treatment period. Three days after the concanavalin A injection (Day 3), rabbits were euthanized and the vitreous bodies were isolated. The protein concentration in the vitreous body was measured to assess the integrity of the blood-retinal barrier. Each value represents the mean + standard error of the mean (n = 6–7). ** *P* <0.01 vs. Sham (Student's *t* test, one-side), †† *P* <0.01, † *P* <0.05 vs. Saline (Dunnett's test, one-side).

Con A induced intraocular inflammation associated with an increase in the intravitreal concentration of proteins at 72 hours after the intravitreal Con A injection (Figure 4, *P* = 0.0057, Student's *t* test, one-side). The concentration of protein in the vitreous body was significantly reduced in rabbits that had received a subcutaneous injection of indomethacin (*P* = 0.0064, Dunnett's test, one-side). After topical bromfenac, the protein concentration was also significantly reduced to approximately the same level as that of the indomethacin-treated rabbits (*P* = 0.026, Dunnett's test, one-side). It was also reduced in diclofenac- and nepafenac-treated animals but the decrease was not significant.

## Discussion

We compared the ability of diclofenac, bromfenac, and amfenac to inhibit COX-1 prepared from human platelets and recombinant human COX-2 expressed in insect cells and also determined their IC_50_. Our results showed that bromfenac was 2.7 to 10 times more potent in inhibiting COX-1 and COX-2 than diclofenac or amfenac, and also the inhibitory activities of these NSAIDs were not selective for COX-1 and COX-2 (Table 1). Earlier studies showed that the IC_50_ of bromfenac was 0.0864 µM for COX-1 and 0.0112 µM for COX-2. The IC_50_ of amfenac was 0.138 µM for COX-1 and 0.00177 µM for COX-2, [23] and it was also 0.25 µM for COX-1 and 0.15 µM for COX-2 [24]. Walters et al. reported that the IC_50_ was affected by the exposure time and/or the species from which the COX enzymes were obtained [23]. In their study, the COX activity was evaluated by sheep-derived enzymes or a combination of sheep and recombinant human enzymes [23], [24]. Because our COX enzymes were of human origin, our results are probably more appropriate for predicting their efficacy and safety in human patients.

In general, halogenation enhances the potency of medicinal compounds (Br^−^ ∼ I^−^ > Cl^−^ > F^−^ > H) [25]. The chemical structures of bromfenac and amfenac are structurally identical with the exception of a bromine atom at the C_4_ position (Figure 1). This alteration may lead to the higher penetrance of bromfenac into ocular tissues, extend its anti-inflammatory activity, and enhance its inhibitory effect on the COXs [26], [27]. Our findings showed that bromfenac had a stronger inhibitory activity on COX-1 or COX-2 than amfenac or diclofenac which is compatible with earlier reports [26]–[29].

We measured the concentrations of diclofenac, bromfenac, or amfenac in retinochoroidal tissues because data were not available on the simultaneously compared pharmacokinetics of these three NSAIDs without including any metabolites after topical applications. Generally, the absorption of drugs through the cornea is dependent upon their physicochemical properties, e.g., octanol-water partition coefficient (Log P), molecular weight, solubility, and ionization state [30]. Molecules of lower molecular weight and moderate hydrophobicity are absorbed better through the cornea. For example, it has been demonstrated that there is a parabolic relationship between the logarithm of the corneal permeability coefficient and Log P in studies on different steroids, and the optimum Log P was 2.9 [31]. In our study, the penetration of topical diclofenac and nepafenac into the aqueous humor or retinochoroidal tissue was better than that of bromfenac (Figure 2). This may be because the molecular weights of diclofenac or nepafenac are less than that of bromfenac (diclofenac: 296.1, nepafenac: 254.3, bromfenac: 334.2) although the hydrophobicity of bromfenac is proportionate in these NSAIDs (diclofenac [32], [33]: 4.31 and 4.5, bromfenac [27], [33]: 2.23 and 3.22). In addition, our results confirmed that nepafenac is immediately converted to its active form, amfenac, in the eye [34].

Although our results showed that the NSAID eye drops were present in the retinochoroidal tissues after topical application (Figure 2B and Table 3), their penetration route was not determined. In an earlier study with ^14^C-labelled bromfenac eye drops in rabbits, high levels of the radioactivity were detected in the cornea, conjunctiva and sclera. Subsequently, the radioactivity was found in the aqueous humor, iris, and choroid and to a lesser degree in the retina. The level was insignificantly lower in the lens and vitreous body [20], [35]. Therefore, it is possible that topical bromfenac can reach the posterior segment by way of the conjunctival/scleral route, viz., conjunctiva → periocular Tenon tissue → posterior sclera → posterior choroid → retina [30], [36]. This hypothesis is supported by the findings that topically applied radiolabeled iganidipine and nipradilol penetrated through the same pathway determined by an autoradiographic method [36], [37].

It was also important that bromfenac had a longer lag time in the retinochoroidal tissues, and the concentration increased from 30 minutes to 2 hours although tears secreted from the lacrimal gland only allow any topical drugs to remain on the cornea surface for about 5 minutes. An earlier study showed that the concentration in each tissue, such as the cornea/conjunctiva, anterior-sclera, posterior-sclera, and retinochoroidal tissue had a second peak following the initial spike after instillation [35]. The second peak in the anterior and posterior sclera was detected 2 hours after the instillation and that in the retinochoroidal tissue was 4 hours. During that period, the cornea/conjunctiva maintained a comparatively high concentration with only a slight decrease. Thus, the drug was most likely delivered from the cornea/conjunctiva to the posterior eye continuously which may make a longer lag time. However, these findings were made in rabbits, and species difference must be considered when applying these results to humans.

The concentration transition model simulating multiple applications of the three test NSAID eye drops found that the concentration in the aqueous humor at the trough level was higher than the IC_50_ for diclofenac, bromfenac, and amfenac (Figure 3A–C). Thus, we conclude that these three eye drops should be effective against inflammations of the anterior segment of the eye as has been found in clinical reports [11]–[13], [38]–[40]. Thus, our model simulating the local concentration based upon the results of a single application was able to support the clinical efficacy of these three NSAID drugs.

Using this model, we found that the concentration of bromfenac in retinochoroidal tissue was higher than the IC_50_ even at the trough level with both two and three applications/day regimen, but the other NSAID eye drops were not (Figure 3D–F, Figure S1). These results indicate that only bromfenac when applied topically will have anti-inflammatory effects on retinochoroidal inflammation induced by intravitreal Con A in rabbits but the other NSAIDs will not (Figure 4).

Kapin et al. reported that 0.5% nepafenac eye drops could significantly inhibit the breakdown of the blood-retinal barrier in a Con A-induced intraocular inflammation model [22]. However, this effect was not found in our study. This difference was probably because they used a 0.5% nepafenac eye drops which is 5 times higher than the concentration commercially available, and they also used a 5 times/day regimen. As a result, the efficacious concentration was not achieved by our protocol of 0.1% nepafenac 3 times a day. Bucolo et al. also reported that topical indomethacin, bromfenac and nepafenac significantly reduced retinal PGE_2_ levels with higher effect of indomethacin and bromfenac in comparison with nepafenac, however, only indomethacin was able to prevent retinal vascular leakage in lipopolysaccharide-injected rats [41]. Their instillation protocol and inflammation model were different from ours, which made it difficult to compare our data directly.

Although anti-VEGF treatment is the gold standard therapy for nAMD at present, it is required to reduce the treatment burden. For that, Gomi et al. and Flaxel et al. independently reported the possible benefit of the adjunctive use of topical bromfenac with the anti-VEGF agents [18], [42]. Because the pharmacokinetics or the local concentrations of NSAIDs after topical application had not been determined, the treatment effect was not fully validated. Our results support the rationale for this treatment. However, further clinical evidence would be required to determine the effect of topically applied bromfenac on retinochoroidal inflammatory diseases.

Con A was used to induce retinochoroidal inflammation because of its easy availability and its well-known characteristics. Con A is a nonspecific inflammatory agent of the lectin group and has a mitogenic effect on T cells and some B cells [43], [44]. It causes a long-lasting inflammatory response which continues with periods of aggravation and alleviation. The effect of an intravitreal injection of retinal-S antigen, rhodopsin, recoverin, or interphotoreceptor retinoid-binding protein have been examined in our preliminary studies. In comparison to them, the Con A model had enough but not devastating inflammation, which was suitable for evaluating dysfunction of the blood-retinal barrier.

For clinical data, a population pharmacokinetics analysis has been widely used, which is suitable for correlating the variations in the drug concentrations among individuals who are the target patient population receiving clinically relevant doses of a drug of interest. It has the advantage to be able to analyze numerous data from small samplings at irregular time points in a large number of individual subjects. Even for that model, numerous data at different time points are necessary to predict an accurate concentration. In our animal study, however, the number of controlled rabbits with the designated sampling time was sufficiently prepared, and thus the present standard pharmacokinetic model was used. A population pharmacokinetics would be necessary for the next step of a clinical study in humans.

There are no data regarding the terminal eliminating phase or mass balance to define the drug bioavailability after NSAIDs are applied by eye drops because the relative contributions of absorption into and clearance from the tissue were not known in this study. Therefore, even though bromfenac was present at the site of interest, it does not necessarily mean it is biologically active. However, it could be possible that biologically active bromfenac would exist at retinochoroidal tissue because the inflammation was significantly inhibited in the Con A model after bromfenac eye drop treatment.

We also did not directly measure the concentration of bromfenac after steady state after multiple instillations. Therefore, the treatment effect of bromfenac in the Con A model was not completely proven. But the concentration of bromfenac in retinochoroidal tissues was reported to be 5.8 ng/mL (7.2 ng/mL × 80.5%, where 80.5% is the proportion of bromfenac in the metabolites) at 8 hours after the last instillation during 21 day-once daily instillation of 0.1% ^14^C-bromfenac [35]. This concentration is similar to the level, 6.44 ng/mL, at 8 hours after the last instillation of our simulation model in the case of 21 day-once daily instillation (data not shown). These limitations should be remembered in interpreting the present results.

In conclusion, our pharmacokinetic results showed that topical bromfenac can be penetrated into the retinochoroidal tissues in high enough concentrations to inhibit COX-2. An inhibitory effect of topical bromfenac on the blood-retinal barrier breakdown was also observed in an experimental retinochoroidal inflammation in rabbits. NSAIDs eye drops have been used for more than 10 years for various anterior segment diseases and its low systemic invasiveness, low cost, and safety have been established. For example, indomethacin has been used for the treatment and prevention of cystoid macular edema related to cataract surgery successfully [11]. However, considering our results and the effect of NSAIDs, NSAIDs eye drops, especially bromfenac, should have therapeutic benefits for retinochoroidal inflammatory diseases more broadly than have been reported.

## Supporting Information

Figure S1 **Simulated bromfenac concentration in retina/choroid in a three times/day dosing and the IC_50_ for COX-2.** The pharmacokinetic profile of bromfenac was calculated on the basis of a three/day dosing at an interval of eight hours to illustrate the pharmacokinetic profile in the same dosing conditions on the Con A model in rabbits (Figure 4). The simulated concentration (solid line) of bromfenac in retinochoroidal tissues with a three/day dosing was also higher than the IC_50_ value for COX-2 (dotted line). The value of the IC_50_ indicated in the figure is referred in Table 1.
(TIF)

Appendix S1 **Detailed analytical condition of LC-MS/MS.**
(PDF)
