# Supplementary figures and images for: Pharmacokinetics and Efficacy of Topically Applied Nonsteroidal Anti-Inflammatory Drugs in Retinochoroidal Tissues in Rabbits
Source: PLoS One. 2014 May 5;9(5):e96481. doi: 10.1371/journal.pone.0096481 (PMC4010472; doi:10.1371/journal.pone.0096481)

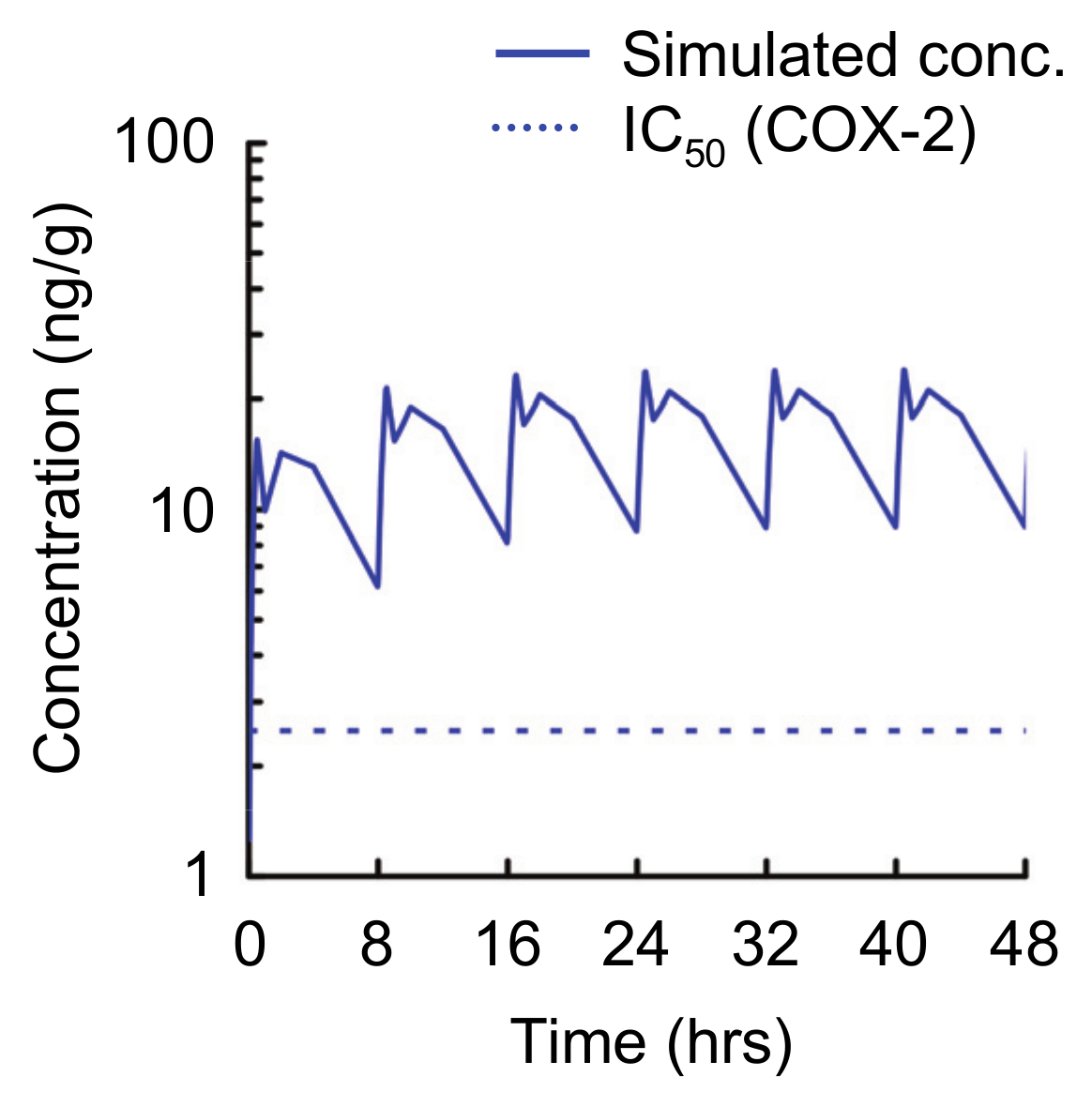

Supplement: Figure S1 — Simulated bromfenac concentration in retina/choroid in a three times/day dosing and the IC50 for COX-2. The pharmacokinetic profile of bromfenac was calculated on the basis of a three/day dosing at an interval of eight hours to illustrate the pharmacokinetic profile in the same dosing conditions on the Con A model in rabbits (Figure 4). The simulated concentration (solid line) of bromfenac in retinochoroidal tissues with a three/day dosing was also higher than the IC50 value for COX-2 (dotted line). The value of the IC50 indicated in the figure is referred in Table 1. (TIF) [file pone.0096481.s001.tif]
