# Supplementary material for: Pharmacokinetics and Efficacy of Topically Applied Nonsteroidal Anti-Inflammatory Drugs in Retinochoroidal Tissues in Rabbits
Source: PLoS One. 2014 May 5;9(5):e96481. doi: 10.1371/journal.pone.0096481 (PMC4010472; doi:10.1371/journal.pone.0096481)
Supplement: Appendix S1 — Detailed analytical condition of LC-MS/MS. (PDF) [file pone.0096481.s002.pdf]

Appendix S1. Detailed analytical condition of liquid chromatography-tandem mass spectrometry (LC-MS/MS).

<u>LC conditions:</u>

LC system: Shimadzu 30A system (Shimadzu Corp., Kyoto, Japan)
Column: ACQUITY UPLC BEH C18 (2.1 × 50 mm, 1.7 μm, Waters Corp., Milford, MA)
Guard column: VanGuard HSS T3 (2.1 × 5 mm, 1.8 μm, Waters Corp.)
Column temperature: 40ºC
Sample cooler temperature: 4ºC
Flow rate: 0.6 mL/min
Injection volume: 50 μL
Mobile phases: A = 0.05% formic acid, B = methanol
Needle wash solvents: 0.05% formic acid, 300 μL → 40% methanol, 300 μL
Gradient: at 0.0 min, 40% B
at 1.0 min, 40% B
at 3.0 min, 80% B
at 4.5 min, 80% B
at 4.6 min, 40% B
at 6.0 min, 40% B

<u>MS/MS conditions:</u>

MS system: QTRAP 5500 system (AB SCIEX, Framingham, MA)
Ionization method: electrospray ionization (ESI, positive mode)
Scan mode: multiple reaction monitoring (MRM)
Turbo gas temperature: 500ºC
Monitoring ion (Q1/Q3, m/z):

bromfenac:	334/288
nepafenac:	255/210
amfenac:	256/210
diclofenac:	296/214
diclofenac-d4:	302/256
